# An ERP study on multiplication and its relationship to phonological processing in children and adults

**DOI:** 10.1007/s00426-024-02036-6

**Published:** 2024-12-06

**Authors:** Heather G. Simpson, Lisa M. Henderson, Silke M. Göbel

**Affiliations:** 1 Department of Psychology, University of Edinburgh, Edinburgh, UK; 2 Department of Psychology, University of York, York, UK; 3 Department of Special Needs Education, University of Oslo, Oslo, Norway

## Abstract

Associations between arithmetic and reading skills suggest that these important abilities may rely, at least in part, on shared neurocognitive processes. It has been argued that retrieval of arithmetic facts may rely on phonological processing; however, very few studies have explored this association using neural indices and whether it manifests similarly in children and adults. Here we examined event related potentials (ERPs) as an indirect neural correlate of arithmetic fact retrieval, and whether variability in ERP modulation is associated with individual differences in phonological processing (verbal working memory, rate of access, and phonological awareness). Arithmetic processing was examined in two samples with different levels of arithmetic expertise: (1) young adults (*n* = 24; *M*_age_ = 21.8 years); and (2) children (*n* = 25; *M*_age_ = 11.2 years). Participants were presented with simple multiplication equations that were correct or incorrect. Significant modulations of the ERPs by correctness were found at posterior electrodes in both samples, however, in different components. In adults a modulation of the P300 was observed, while for children the N400 response was modulated. For both children and adults, the size of the ERP modulation in posterior electrodes was associated with individual differences in verbal working memory. These results highlight an important distinction between behavioral outcomes and their underlying neurocognitive mechanisms. Additionally, they provide insight into how arithmetic processing evolves over the course of development.

## Introduction

Every day we rely on numerical problem-solving to make decisions, such as whether it is more cost-effective to buy the pre-packaged or loose potatoes, or establishing when we need to leave the house to keep an appointment. Not only is numeracy a fundamental life skill, but mastery and efficient retrieval of basic arithmetic facts form the basis from which other higher-order mathematical concepts develop (Wong & Evans, 2007). Multiplication tables tend to be learned verbally, and it has been suggested that these facts are stored in semantic memory as verbal codes (Dehaene et al., 2003). Retrieval of arithmetic facts may therefore rely on the cognitive and neural processes that also support language and literacy, including phonological processing (Pollack & Ashby, 2018). While there is behavioral evidence for an association between mathematical achievement and phonological processing (e.g., De Smedt & Boets, 2010; De Smedt et al., 2010; Hecht et al., 2001; Schleepen et al., 2016), few studies have examined how this association is supported by the brain as arithmetic skills develop. Brain imaging studies have highlighted a network of interconnected brain regions supporting arithmetic, involving frontal and parietal regions (Menon, 2011, 2015; Peters & De Smedt, 2018). This network, as well as brain areas supporting phonological processing, are influenced by brain maturation and experience (Lyon & Rumsey, 1996), and undergo functional and structural changes during development (Bunge & Wright, 2007; Houston et al., 2014; Peters & De Smedt, 2018).

In the present study we use event-related potentials (ERPs) to investigate the neurocognitive mechanisms underlying arithmetic fact retrieval, and their association with phonological sub-processes, in 9- to 12-year-old children and adults, as these skills become increasingly automatized. We have chosen ERPs as a method for two reasons. First, from behavioral data it is well known that with increasing expertise arithmetic processing becomes faster (e.g., De Smedt, 2016). EEG has excellent temporal resolution (Luck, 2014) and thus can provide information about arithmetic and cognitive processing as it unfolds with millisecond precision. Second, several studies using ERPs during language processing (Boudewyn, 2015) have added to our understanding of the influence of individual differences, for example in working memory capacity (Nakano, Saron & Swaab, 2010), on how the brain processes language. Inspired by this literature, we chose ERPs to investigate the relationship between individual differences in phonological skills and variability in how the brain processes arithmetic problems.

### Phonological processing and arithmetic

Phonological processing refers to the ability to recognize and manipulate the speech sounds that make up written and oral information. Wagner and Torgesen (1987) differentiated three phonological sub-processes: phonological awareness, phonetic recoding in working memory, and the rate of access to phonological codes. It is well established that phonological processing is essential for many aspects of language and literacy development, including reading, spelling and vocabulary acquisition, but evidence is also converging to suggest that children may utilize phonological processing abilities to represent, retrieve, and manipulate arithmetic knowledge (e.g., Peng et al., 2020; Yang et al., 2022).

*Phonetic recoding in working memory, or verbal working memory (WM)*, enables an individual to encode and temporarily maintain sound-based representations in mind (Baddeley, 1986), a skill essential to mental arithmetic. During the formative years of schooling, children rely on verbal WM during the verbal rehearsal of multiplication tables, as they hold the sum, its components and the answer in mind (e.g., Rasmussen & Bisanz, 2005). It is through this process that an association is created between the operands and solution. With experience these associations are more likely to be stored in long-term memory and accessed through semantic retrieval (e.g., Geary et al., 2004; Soltanlou et al., 2015). While verbal WM initially seems to facilitate the encoding of arithmetic facts, once these facts become more stable in long-term memory, verbal WM facilitates more complex arithmetic problem-solving, by maintaining information about the arithmetic problem in mind while further calculations are performed (Cragg et al., 2017; Fürst & Hitch, 2000).

The *rate of access to phonological information* in long-term memory (henceforth referred to as rate of access), concerns the efficiency with which one can access phonological codes within the semantic memory (e.g., Wagner & Torgesen, 1987). It has been suggested that before solving an arithmetic problem (e.g., 4 × 6 =), written symbols are first encoded into phonological codes (i.e., “four times six equals”), and thus the more efficiently an individual can access these phonological codes, the quicker they might retrieve the phonologically-based answer from long-term memory (Hecht, 1999). One study found that rate of access in nursery school children predicted concurrent arithmetic fluency after accounting for nonverbal IQ, phonological short-term memory and speed of processing (Cui et al., 2017). Similarly, performance in rapid automatized naming (RAN) tasks, used to assess rate of access in kindergarten and Grade 1, predicted arithmetic fluency in Grade 2 and 3 (Koponen et al., 2016). However, this relationship might be age-dependent, because a study with young adults failed to find an independent association between rate of access and arithmetic performance (De Smedt & Boet, 2010).

*Phonological awareness* refers to knowledge of the sound structure of speech (e.g., Wagner & Torgesen, 1987). It involves identifying the sounds within words and synthesizing these speech segments. Research into the relationship between phonological awareness and arithmetic problem-solving has yielded mixed result, with some researchers suggesting that any association between these factors may be the result of other mediating variables, such as verbal WM (e.g., Hecht et al., 2001; Landerl et al., 2009). Phonological awareness in 9- to 11-year-olds, assessed using a phoneme deletion task, was found to be uniquely and concurrently associated with multiplication fact retrieval, independent of verbal WM and intellectual ability (De Smedt et al., 2010). A later study by the same group failed to replicate this association in children (Schleepen et al., 2016), but a strong link was reported between phonological awareness and arithmetic fact retrieval in students with a history of developmental dyslexia (De Smedt & Boets, 2010).

In summary, whilst there is evidence for an association between arithmetic performance and verbal WM, rate of access appears to be more important during childhood than in adulthood, and whether there is an association between arithmetic and phonological awareness remains unclear. To investigate these associations further, we examined an electrophysiological measure which has been used to assess both arithmetic and linguistic processing.

### Event-related potentials

First described in psycholinguistic research (Kutas & Hillyard, 1980), the N400 event-related potential (ERP) has helped to advance our understanding of how language is encoded by the brain. In a typical N400 paradigm, participants are presented with sentences which are either semantically congruent, e.g., “*I take my coffee with ****sugar***”, or incongruent, e.g., “*I take my coffee with ****salt***”. Approximately 400 ms after the presentation of the target word, i.e., *salt/sugar*, a negative deflection in the ERP can be observed, which is enhanced over central-parietal regions to incongruent compared to congruent targets. Not only is the N400 influenced by the semantic congruity of a sentence, but the amplitude is modulated by the degree of semantic relatedness. This means that the less likely a target word is, the larger the N400 amplitude (e.g., Kutas & Hillyard, 1984). This has led researchers to conclude that the N400 is an index of semantic access, such that the N400 amplitude is reduced when information has already been primed by prior stimuli or contextual cues. Conversely, the N400 amplitude is enlarged when a target is encountered that is out of context and requires new information to be activated (Federmeier, 2022).

A modulation of the N400 component has also been reported during arithmetic tasks. Using a multiplication verification task, Niedeggen and colleagues found that adults showed a larger N400 over centro-parietal regions of the scalp when target solutions were incorrect (e.g., 8 × 2 = ***32***) compared to when they were correct (e.g., 8 × 2 = ***16***) (Niedeggen & Rӧsler, 1999; Niedeggen et al., 1999). Additionally, they found that the N400 amplitude was modulated by the degree of table-relatedness: incorrect solutions which were unrelated to both operands (e.g., 6 × 4 = ***25***) were followed by a larger amplitude than incorrect solutions which were multiples of one or both operands (e.g., ***28*** or ***36***), especially at anterior electrode sites (Niedeggen & Rӧsler, 1999). Thus, it has been suggested that the arithmetic N400 effect could be an index of the semantic access, reflecting the additional cortical activation required to process partially-primed or unprimed solutions (Niedeggen et al., 1999). Specifically, while table-related solutions may already be partially activated by one/more of the operands, a table-unrelated solution first might require cortical activation before this target solution can be rejected (Stazyk et al., 1982). Notably, this electrophysiological effect is in contrast to the behavioral effect of relatedness, given that participants generally take longer and are less accurate in verifying table-related than table-unrelated trials (e.g., Campbell, 1987; Stazyk et al., 1982).

More recent research has, however, questioned this traditional interpretation of an arithmetic N400 in adults (e.g., Dickson & Federmeier, 2017; Dickson & Wicha, 2019; Jasinski & Coch, 2012). Specifically, it has been suggested that the N400 effect (calculated as the difference between the N400 to correct vs. incorrect solutions) masks a slightly earlier positive-going deflection in adult’s ERP to correct solutions, approximately 300 ms after solution presentation (P300; Jasinkski & Coch, 2012). Typically seen in tasks assessing information-processing, attention and memory (Polich, 2012), a P300 ERP is proposed to reflect a participant’s response to the detection of a target, in this case, the correct solution (Jasinski & Coch, 2012). Moreover, problem size appears to modulate the P300 of both correct and incorrect solutions, with smaller problem sizes (e.g., 2 × 3) producing larger amplitudes relative to larger problems (e.g., 8 × 7; Dickson & Wicha, 2019). On this basis, Dickson and Wicha suggest that a larger P300 may reflect participants’ confidence and the ease with which targets are verified.

### Developmental differences in the processing multiplication facts

The neurocognitive processes underlying multiplication fact retrieval are likely to evolve with age and experience. Using a multiplication verification paradigm, Grenier et al. (2020) found that a P300 response was observed in adults, while an N400 was seen in children’s ERPs. This finding potentially highlights fundamental age-related differences in the electrophysiological responses during multiplication, and suggests that the development of the neural network underpinning fact retrieval may occur more gradually than previously reported (Grenier et al., 2020). Adults’ experience is reflected in the efficiency with which they recognize correct solutions, i.e., processing multiplication facts at a relatively superficial level (Dickson et al., 2022) as targets. In contrast, the N400 response by children demonstrates their relative inexperience, as they process the meaning of the stimuli before selecting a response (Grenier et al., 2020). Specifically, the N400 amplitude generated by children is believed to reflect ease of semantic access (Dickson et al., 2022), as multiplication solutions are activated within semantic memory networks (Niedeggen & Rӧsler, 1999; Niedeggen et al., 1999). Given that correct solutions are more accessible, the N400 amplitude is reduced (i.e., less negative) on correct trials relative to incorrect trials.

Like the P300, the N400 response also appears to be modulated by specific features of the multiplication problems. Recent evidence by Dickson et al. (2022) indicates that the N400 in children is sensitive to problem size, although the effect was specific to correct solutions, with a reduced N400 amplitude found to smaller relative to larger problems. Whether the N400 is modulated by table-relatedness in a developmental sample remains to be seen. Children show higher error rates and longer reaction times for table-related than table-unrelated incorrect solutions (e.g., Koshmider & Ashcraft, 1991; Soltanlou et al., 2015), and thus it seems plausible that children’s ERPs in response to incorrect solutions may be affected by whether the incorrect solutions are table-related or table-unrelated.

### The present study

This study had two overarching aims. First, we assessed arithmetic processing, as indexed by the N400 and/or P300, in experienced multipliers, i.e., young adults, and comparatively novice multipliers, i.e., 9- to 12-year-old children. By the end of Year 4 (9 years of age), children in England are expected to be proficient in their multiplication tables up to 12 × 12 (DfE, 2013). However, because these skills are relatively newly acquired and neural networks supporting arithmetic processing are still developing, we expect arithmetic retrieval to be less efficient in this group relative to adults. Moreover, we aimed to extend previous research to examine the effect of table-relatedness. While an effect of relatedness has been reported for the N400 in adults (e.g., Niedeggen, et al., 1999), it has not been found for the P300 (Dickson & Federmeier, 2017). Examining this effect in both adults and children, offers insight into the development of neurocognitive processes supporting arithmetic processing, specifically when overcoming interference from related (but incorrect) information.

Second, we examined how neural indices of arithmetic processing in young adults and 9- to 12-year-old children relate to phonological processing, i.e., phonological awareness, rate of access, and verbal WM. We expected a relationship between individual differences in phonological processing and ERP amplitudes over posterior electrodes for two reasons. First, both the P300 and N400 components tend to be most prominent (Grenier et al., 2020; Polich, 2012) over posterior electrodes and second, both multiplication and phonological processing have been shown to activate the inferior parietal lobule (e.g., Andin et al., 2015; Pollack & Ashby, 2018).

## Study 1: Adult multipliers

In Study 1 we assessed the association between arithmetic retrieval and phonological processing in young adults, i.e., experienced multipliers. Based on previous findings (Niedeggen & Rӧsler, 1999; Prieto-Corona et al., 2010), we expected a significant arithmetic N400 effect over centro-parietal regions, i.e., a larger N400 in response to incorrect than correct multiplication solutions. However, in line with recent literature (e.g., Dickson & Federmeier, 2017; Dickson & Wicha, 2019; Jasinski & Coch, 2012), a P300 response was not ruled out. Secondly, we expected a significant effect of relatedness in (a) adult behavioral data, i.e., greater accuracy and faster responses to table-unrelated than table-related solutions; and (b) adult ERP data, i.e., we expected that the ERP amplitude to incorrect solutions would be modulated by table-relatedness. Finally, because a significant association has been reported between verbal WM and the semantic N400 amplitude in adults (Kim et al., 2018), we predicted an association between verbal WM and ERP amplitudes in posterior electrodes.

### Material and methods

#### Participants

Thirty-six young adults from the University of York gave written consent, and were compensated for their time. Apart from one German and one Serbian student, all participants were native English-speakers. Participants had normal or corrected-to-normal vision, and no history of brain injury. Once inaccurate responses were removed, only participants who had more than 50% of EEG trials remaining after artifact rejection were included, which excluded nine participants from the final analysis. An additional three participants were excluded due to a fault in the reference electrode, and one participant had missing behavioral data, leaving a final sample of 24 right-handed adults (*M*_age_ = 21.8 years, *SD* = 3.19, range: 18–31 years, 12 female). The study received ethical approval by the Ethics Committee at the Department of Psychology, University of York.

#### Materials

##### Phonological processing

1. *Phonological Awareness* was assessed using the Phonemic Decoding Efficiency (PDE) subtest of the Test of Word Reading Efficiency (TOWRE; Torgesen et al., 1999). During this timed task (45-seconds), participants are required to sound out a list of non-words. One point was given per correct response (maximum score = 63). The test-retest reliability reported in the test manual is .83 for children aged 9-12 years and .94 for adults.
2. *Verbal Working Memory (WM)* was assessed using the Memory for Digits subtest from the Comprehensive Test of Phonological Processing (CTOPP; Wagner et al., 1999). Each item consists of a series of digits, spoken at a rate of two digits per second. Starting with a two-digit sequence, sequences increase in length to a maximum of eight digits (maximum: 21 items). Participants were required to immediately recall the sequence of digits, and the subtest was discontinued after three successive errors. One point was given per item which was error-free. The test-retest reliability reported for the Memory for Digits in the test manual is .81 for adults and .74 for children aged 9-12 years.
3. *Rate of Access* was assessed using the Rapid Letter Naming subtest of the CTOPP (Wagner et al., 1999). Participants were required to rapidly name randomly arranged letters. Examinees were timed as they read out the letters as fast as possible. Scores were calculated as the time taken to name all the letters, meaning that a lower score was associated with better rate of access. The test-retest reliability reported in the test manual is .86 for adults and .94 for children aged 9 to 12 years.

##### Non-verbal reasoning

The Matrix Reasoning subtest from the Wechsler Abbreviated Scale of Intelligence (WASI-II) was used to estimate general cognitive ability (Wechsler & Hsiao-pin, 2011). This task consists of 30 matrices, in which one of the squares is missing. Participants were required to select one of five options, to complete the pattern. Testing was discontinued after three consecutive scores of zero, and one point was awarded for every correct response (maximum score: 30). The test–retest reliability reported in the test manual is 0.72 for adults and 0.76 for children.

##### Mathematical computation

The math computation subtest of the Wide Range Achievement Test (WRAT-4) was used to assess mathematics achievement (Wilkinson & Robertson, 2006). Participants were given 15 min to complete as many of the 40 items as possible. All participants answered at least five questions correctly and were automatically credited with 15-points from the Oral Math sub-test (maximum score: 55). The test–retest reliability reported in the test manual is 0.88 for adults and 0.77 for 9 to 12 year-old children.

##### Computerized multiplication task

A multiplication verification task, during which EEG was recorded, required participants to verify whether the proposed target solution of each multiplication item was correct. Stimuli consisted of multiplication problems created from all possible combinations of two single-digit operands (number range: 2–9), with ties excluded (e.g., 4 × 4) (McCloskey, 1992). Fifty-six unique combinations (e.g., 2 × 3) were generated from these operands, and presented eight times resulting in 448 experimental trials. Each problem was presented four times (50%) with the mathematically correct target solution (e.g., 2 × 3 = 6), and four times (50%) with an incorrect solution. For each problem, two incorrect trials had table-related solutions (e.g., 2 × 3 = 9) and two incorrect trials had solutions that were not table-related (e.g., 2 × 3 = 11). Table-related problems were constructed so that half were in close proximity to the correct solution [(a ± 1) x b], and half were further away from the correct product [(a ± 3) x b]. Table-unrelated problems were constructed by adding or subtracting two from the table-related products. One half of the table-unrelated products were close to the correct answer [(a ± 1) x b] ± 2, and the other half were further away from the correct product [(a ± 3) x b] ± 2. Correct and incorrect problems were matched overall on problem size, and table-related and -unrelated problems were matched overall on problem size and overall distance from the correct solution.

**Fig. 1:**
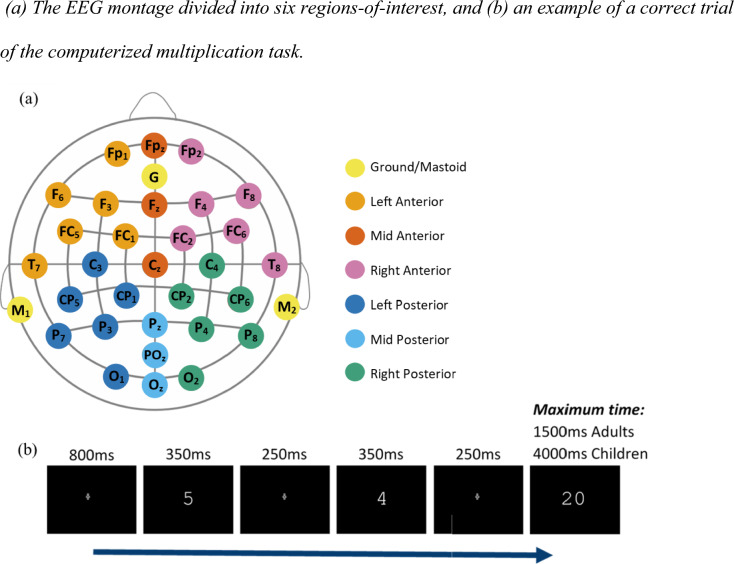
**a** The EEG montage divided into six regions-of-interest, and **b** an example of a correct trial of the computerized multiplication task

After eight practice trials, participants verified 28 blocks of 18 multiplication problems, balanced to include an even distribution of problem types (448 assessed trials, 56 fillers). The first two problems within each block were filler trials. Problems for the filler trials were created following the same procedure as described above with one change: b was always 11 or 12. All filler trials were excluded from analysis to allow the EEG signal to stabilize following breaks. Participants viewed blocks sequentially on the screen, in the same fixed order. The sequence of each problem was: a fixation cross (font size 18, displayed for 800 ms), the first operand (350 ms), a fixation cross (250 ms), the second operand (350 ms), another fixation cross (250 ms), and finally the target solution (max: 1500 ms) (see Fig. 1b)^1^. Participants were instructed to respond as quickly as possible, using “F” and “J” keys on the keyboard, to indicate whether the proposed solution was correct or incorrect (allocation of keys was reversed for half of the participants). Reaction time was calculated from the onset of the target solution until the participant responded.

#### Procedure

In a quiet room, participants were assessed individually on a battery of behavioral assessments in the same fixed order. The session lasted a maximum of 2.5 h and was concluded with the computerized multiplication task during which EEG was recorded.

##### EEG testing

EEG recording took place with the WaveGuard system (ANT Neuro, The Netherlands). Data were recorded from 32 silver-silver chloride electrodes. Signals were amplified using a high-speed 64-channel amplifier, with a sampling rate of 500-Hz, and recorded on a Z230 HP Computer using Asalab 4.9.1. Electrodes were arranged in a 10–20 system (see Fig. 1a) using the following channels: Fp1, Fpz, Fp2, F7, F3, Fz, F4, F8, FC5, FC1, FC2, FC6, M1, T7, C3, Cz, C4, T8, M2, CP5, CP1, CP2, CP6, P7, P3, Pz, P4, P8, POz, O1, Oz, O2. All electrodes were referenced online to the left mastoid (M1). The ground electrode was placed anterior to the Fz channel. To measure eye movements the vertical and horizontal electrooculogram (VEOG/HEOG) was measured above the eyebrow and below the eye, respectively. We ensured that the impedance levels fell below 8kΩ for all electrodes before the recording.

##### Computerized multiplication task

Stimuli were presented on a Samsung SyncMaster 2333SW 23-inch LCD screen (refresh rate: 60 Hz; resolution: 1280 × 1024 pixels), using E-prime 2.0 (Psychology Software Tools, Inc., USA). Stimuli appeared centrally (black background, grey size 64 “courier new” font) at a visual angle of 1.3° × 1°.

### Data analysis

EEG data were pre-processed offline using BrainVision Analyzer 2 (Brain Products GmbH, Germany). Raw data were re-referenced (to the average of M_1_ & M_2_), and a bandpass filter (0.1 to 20 Hz, 12 dB/oct, 50 Hz notch) applied (Duncan et al., 2009). Due to errors in coding, three unrelated trials were excluded. All remaining experimental trials that were responded to correctly were segmented (time window for each trial: − 200 ms to 1500 ms). Baseline correction was applied, using a 200 ms pre-stimulus window (− 200 ms to 0 ms). Automatic artifact rejection was performed over a 950 ms interval (− 200 to 750 ms) using the criteria set out by Kappenman et al. (n.d.) [gradient: 75 µV/ms, amplitude: ± 100 µV, minimum–maximum difference: greater than 150 µV/200 ms or less than 0.5 µV/50 ms]. After excluding trials containing artifacts, an average of 326 trials (81%) remained per participant (*SD* = 53; range: 193–407). This consisted of an average of 164 correct trials (81%, *SD* = 27; range: 106–205); 79 related trials (82%, *SD* = 14, range: 46–100); and 83 unrelated trials (80%, *SD* = 15, range 41–106) per participant.

**Fig. 2:**
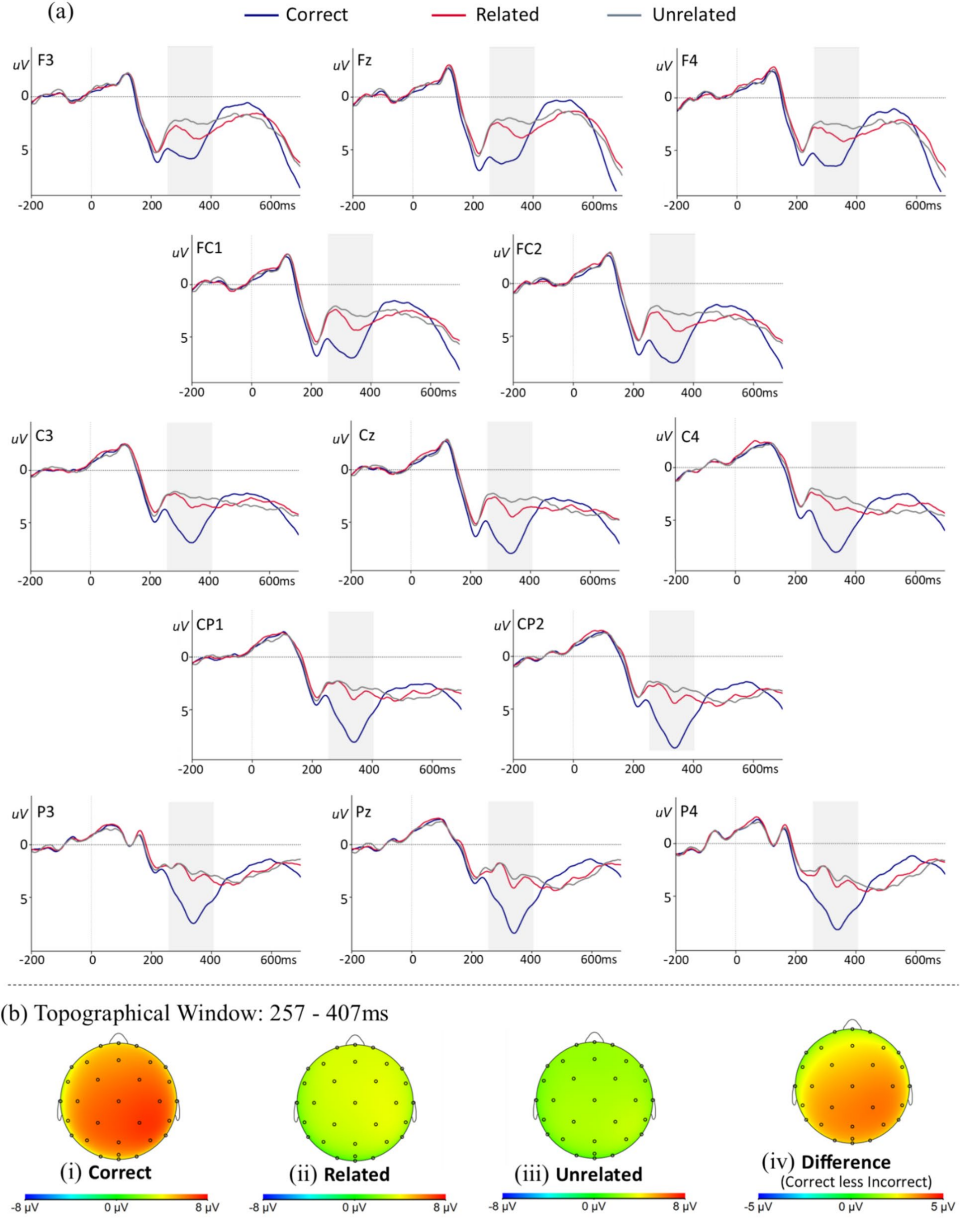
**a** Grand average ERPs on adult’s correct (blue), related (red) and unrelated (grey) conditions at electrodes sites across the scalp. The shaded area highlights the time-window (257–407 ms) of the analyses (negative voltage plotted up). **b** Topographical plots of the grand average for: (i) correct, (ii) related and (iii) unrelated conditions; and (iv) the P300 effect

Average ERPs were generated for each participant at every electrode for each condition, time-locked to the onset of the target solution. Additionally, a grand-average ERP waveform was generated for each condition and electrode. Visual inspection of the grand-average ERP waveforms (Fig. 2a) revealed a negative deflection 250–300 ms after solution presentation, which was more prominent for the incorrect (related and unrelated) than the correct solutions. This was followed by a positive peak approximately 350 ms after solution presentation, which was larger in for the correct than incorrect solutions. In contrast to our hypothesis, there was no clear indication of an N400. However, our data provide evidence of a P300 which was more prominent for correct than incorrect solutions. Therefore, further analyses were limited to the P300 component.

Time windows for the analysis were based on visual inspection of the grand-average waveforms, and guided by previous literature (Dickson et al., 2018; Grenier et al., 2020; Jasinski & Coch, 2012). Using a window of 250–500 ms, the overall P300 peak amplitude was found at 332 ms (SD = 28 ms) [for the correct condition: 329 ms (*SD* = 25 ms), incorrect: 335 ms (*SD* = 31 ms), related: 336 ms (*SD* = 30 ms), unrelated: 340 ms (*SD* = 34 ms)]. Using a 150 ms window (257–407 ms, i.e., 75 ms on either side of the peak latency) we then extracted mean amplitudes for each participant at each electrode and for each condition (correct, incorrect, related, unrelated) in BrainVision Analyzer.

Exported data were averaged across the channels for each ROI for each participant by condition. Averages were calculated for six electrode groups (ROIs): left-anterior (Fp_1_, F_3_, F_7_, FC_1_, FC_5_, T_7_), left-posterior (C_3_, CP_1_, CP_5_, P_3_, P_7_, O_1_), midline-anterior (Fp_z_, F_z_, C_z_), midline-posterior (P_z_, PO_z_, O_z_), right-anterior (Fp_2_, F_4_, F_8_, FC_2_, FC_6_, T_8_), and right-posterior (C_4_, CP_2_, CP_6_, P_4_, P_8_, O_2_) (Fig. 1a). In the correlational analyses we investigated whether there was a significant association between individual differences in the phonological skills and the average amplitudes in each of the three posterior ROIs (left-posterior, midline-posterior, right-posterior).

### Statistical analysis

The data were analyzed using IBM SPSS Statistics (v25). For the computerized multiplication task percentage accuracy and mean RT (from target presentation to response) were calculated for each condition. To eliminate extreme scores from RT data, a sequential filtering procedure was applied and repeated twice. RTs outside ± 3SD of a participant’s mean were discarded (trials retained: 97%), and the mean and standard deviation recalculated (see Bahnmueller et al., 2016). Analysis of variance (ANOVA) was used to assess whether condition had a significant effect on accuracy and RT. Helmert contrasts were used to examine statistically significant effects. These were set up to account for unequal trial numbers within our conditions (contrast 1: correct vs. incorrect (related and unrelated) | contrast 2: related vs unrelated).

A three-way repeated measures ANOVA was run on the mean P300 amplitude, with factors of condition (correct, related, unrelated), hemisphere (left, midline, right) and caudality (anterior, posterior). Helmert contrasts were used to examine significant main and interaction effects related to Correctness (correct vs incorrect trials) or Relatedness (related vs unrelated trials). Pearson’s correlations were calculated to examine the association between participants’ scores on the three phonological sub-processes and their average amplitude of the P300 for the posterior ROIs. Because raw scores were used in the correlations, age was entered as a covariate, in addition to nonverbal reasoning. To control Type I error rate, a Benjamini–Hochberg false discovery rate (FDR; Benjamini & Hochberg, 1995) was applied within each phonological sub-process.

## Results

The mean, standard deviation and range of the behavioral measures are displayed in Table 1. A correlation matrix of these outcomes is provided in the Appendix (Table 4).

**Table 1:** Mean (M) scores on behavioral measures, with standard deviation (SD) and range

	Adults (Study 1)			Children (Study 2)		
	*M*	*SD*	Range	*M*	*SD*	*Range*
Nonverbal Reasoning^a^	59.50	8.37	40–71	53.76	10.54	28–72
Math Computation^b^	105.21	11.86	85–129	110.84	16.56	88–143
Phonological Awareness^b^	108.21	9.67	92–120	108.36	13.67	75–131
Verbal Working Memory^c^	12.54	1.64	10–16	11.80	3.00	5–17
Rate of Access^c^	9.13	2.42	2–13	8.64	2.29	6–15

^a^T-Score (40–60 average range)

^b^Standard score (85–115 average range)

^c^Scaled score (8–12 average range)

### Behavioral Data

On the computerized multiplication task, adults achieved an average accuracy rate of 89.58% (*SD* = 4.22%, range: 79–96%). There was a significant effect of Condition on accuracy, *F*(1.5, 35.6) = 16.63, *p* < 0.001, *ɳ*^*2*^_*p*_ = 0.42. Planned contrasts indicated that accuracy did not differ significantly between correct (89.58%, *SD* = 5.32%) and incorrect (88.71%, *SD* = 5.19%) conditions [*F*(1, 23) = 0.37, *p* = 0.55, *ɳ*^*2*^_*p*_ = 0.016], but participant accuracy was significantly higher on unrelated (92.10%, *SD* = 4.20%) relative to related (85.67%, *SD* = 6.68%) trials [*F*(1, 23) = 52.51, *p* < 0.001, *ɳ*^*2*^_*p*_ = 0.70].

On average, participants took 586 ms (*SD* = 98 ms, range = 408–805 ms) to respond to target solutions. There was a significant effect of condition on reaction time, *F*(2, 46) = 46.91, *p* < 0.001, *ɳ*^*2*^_*p*_ = 0.67. Planned contrasts revealed that participants were significantly faster when responding to correct (562 ms, *SD* = 98 ms) relative to incorrect solutions (613 ms, *SD* = 103 ms) [*F*(1, 23) = 54.61, *p* < 0.001, *ɳ*^*2*^_*p*_ = 0.704]; and to unrelated (596 ms, *SD* = 95 ms) relative to related solutions (630 ms, *SD* = 111 ms) [*F*(1, 23) = 33.15, *p* < 0.001, *ɳ*^*2*^_*p*_ = 0.59], see also Table 5 in the Appendix.

Individual differences in adults’ behavioral performance on the arithmetic verification task was not significantly correlated with any of the phonological measures (see Appendix, Table 6).

### Event Related Potentials

#### Effect of correctness

A three-way repeated-measures ANOVA on mean P300 amplitudes revealed a significant main effect of condition, *F*(2, 46) = 41.62, *p* < 0.001,* ɳ*^*2*^_*p*_ = 0.64. Correct solutions (4.98 µV, *SD* = 2.44 µV) had a significantly larger P300 amplitude (*p* < 0.001) relative to incorrect solutions (2.38 µV, *SD* = 2.10 µV). While, the P300 amplitude did not differ by caudality (*p* = 0.54), an effect of correctness was evident in the interaction between condition and caudality, *F*(1, 23) = 17.99, *p* < 0.001, *ɳ*^*2*^_*p*_ = 0.44. Specifically, a larger P300 amplitude was seen in posterior (5.14 µV, *SD* = 2.84 µV) relative to anterior (4.83 µV, *SD* = 2.67 µV) ROIs in the correct condition, while the opposite pattern was seen in the incorrect conditions with larger amplitudes to anterior (2.69 µV, *SD* = 2.70 µV) relative to posterior (2.06 µV, *SD* = 2.23 µV) ROIs. The P300 amplitude also differed between hemispheres, *F*(2, 46) = 7.43, *p* = 0.002, *ɳ*^*2*^_*p*_ = 0.24, and a significant interaction was seen between condition and hemisphere,* F*(2.27, 52.28) = 5.18, *p* = 0.007, *ɳ*^*2*^_*p*_ = 0.18, with a larger difference in P300 amplitude between left and midline ROIs in the correct (0.78 µV) than in the incorrect (0.21 µV) condition (*p* < 0.001). There was also a significant three-way interaction between condition, caudality and hemisphere, *F*(2.45, 56.34) = 3.83, *p* = 0.020,* ɳ*^*2*^_*p*_ = 0.14. This interaction was primarily driven by differences between the incorrect trials.

#### Effect of relatedness

The P300 amplitude did not differ significantly between related (2.62 µV, *SD* = 2.19 µV) and unrelated (2.14 µV, *SD* = 2.01 µV) solutions. An effect of relatedness was however evident in caudality differences, *F*(1, 23) = 4.87,* p* = 0.038,* ɳ*^*2*^_*p*_ = 0.18. A similar P300 amplitude was seen for related and unrelated solutions over posterior ROIs (difference: 0.19 µV), while over the anterior ROIs the P300 amplitude was significantly larger to related than unrelated solutions (difference: 0.77 µV). Finally, there was also a significant three-way interaction involving relatedness. Planned contrast revealed that caudality differences were largest at the midline relative to the left (*p* = 0.018) and right (*p* = 0.025) hemispheres, however this effect was more pronounced for related (0.83 µV) than unrelated (0.53 µV) solutions.

In summary, the P300 amplitude was larger for correct solutions in posterior ROIs, but larger for incorrect solutions in anterior ROIs, and an effect of relatedness was most notable at the anterior-midline ROI.

**Fig. 3:**
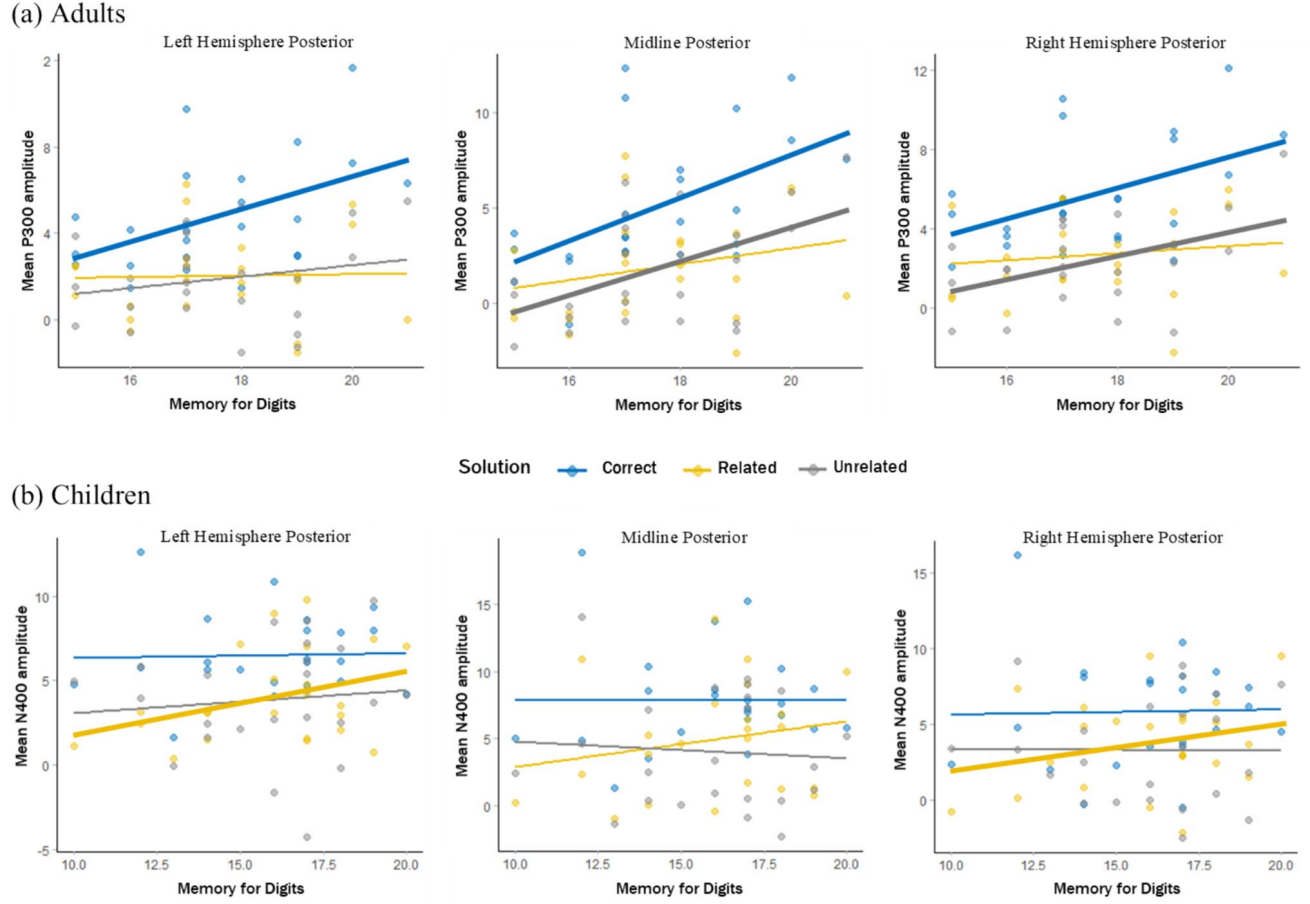
Scatterplots depicting the relationship between verbal WM and **a** P300 amplitude in adults, and **b** N400 amplitude in children over posterior ROIs on the correct (blue), related (yellow) and unrelated (grey) solutions. Bold lines indicate significant relationships

Neither phonological awareness nor rate of access was significantly associated with the P300 amplitude in any ROIs (see Table 7). However, better verbal WM was associated with larger mean amplitudes of the P300 after correct solutions over left-posterior and mid-posterior ROIs, and with larger mean amplitudes of the P300 after unrelated incorrect solutions over the mid-posterior ROIs (see Table 2 & Fig. 3). None of the correlations for the anterior ROIs reached significance.

**Table 2:** Pearson’s correlation coefficients between verbal working memory and the mean amplitude of the P300 after correct, unrelated and related solutions across regions-of-interest (controlling for age and nonverbal reasoning)

		Correct			Unrelated			Related		
ROI		Left	Mid	Right	Left	Mid	Right	Left	Mid	Right
Anterior	*r*	0.153	0.216	0.220	– 0.178	– 0.055	0.072	– 0.171	– 0.105	– 0.072
	*p*	(0.498)	(0.335)	(0.325)	(0.429)	(0.808)	(0.749)	(0.446)	(0.642)	(0.751)
Posterior	*r*	0.523*	0.528*	0.466	0.288	0.545*	0.482	0.034	0.234	0.126
	*p*	(0.013)	(0.011)	(0.029)	(0.194)	(0.009)	(0.023)	(0.882)	(0.295)	(0.576)

^a^ Raw Score; * p < .05 after Benjamini–Hochberg false discovery rate correction

## Study 2: Child multipliers

In study 1 we found evidence of a P300 in the adult data that was influenced by the correctness and relatedness of arithmetic problems. Furthermore, we established that adults with better verbal WM show a larger P300 amplitude in response to correct solutions and incorrect (unrelated) solutions. This pattern might be different when multiplication facts are relatively newly acquired and the semantic memory network is still developing. In study 2 we addressed this by testing 9- to 12-year-old children.

Based on previous findings (Grenier et al., 2020; Prieto-Corona et al., 2010), we expected to find an N400 for children. If the N400 reflects activation of an underlying multimodal memory system, we should find an association between the N400 and verbal WM in children. However, since children are less experienced multipliers and their arithmetic fact networks are still developing, we anticipated that our sample of children would be less efficient at processing arithmetic facts than our adult sample, and that children may draw more heavily on other phonological sub-processes, such as rate of access and phonological awareness, which strongly predict word recognition early in development (e.g., Catts et al., 2015).

To our knowledge, there have been no previous examinations of the effect of relatedness on the N400 amplitude during arithmetic in children. Behavioral data suggest that the effect of relatedness is present only when the network of arithmetic facts is fully established (e.g., Ashcraft, 1992). Effects of relatedness on reaction time and accuracy have been found in children aged 8- and 9-years with strong mathematical skills, and seem to be reliably present in most 10- and 11-year-olds (Koshmider & Ashcraft, 1991; Megias et al., 2015). While evidence of the relatedness effect in children has been reported in behavioral studies, it remains to be seen whether this effect already influences children’s N400 response.

### Method

#### Participants

Forty children (aged 9–12 years) were recruited. Informed consent was obtained from the children and their parents. All children were native English-speakers, with no history of mental illness or brain injury, and normal or corrected-to-normal vision. One left-handed child, one child with missing EEG data and one child with low accuracy were excluded. An additional 12 children were excluded because more than 50% of their EEG trials contained artifacts. The final sample consisted of 25 right-handed children (*M*_age_ = 11.2 years, *SD* = 0.93, range: 9–12 years, 13 female).

#### Materials and methods

The same standardized assessment measures as in Study 1 were used.

##### Computerized multiplication task

Children also completed the multiplication verification task, during which EEG was recorded. However, stimuli consisted of a subset of the items used in study 1. Multiplication problems were created from all possible combinations of two single-digit operands 2–6, with ties excluded (e.g., 4 × 4) following the same procedures as described in study 1. Twenty unique combinations of these operands (e.g., 2 × 3) were presented eight times resulting in 160 experimental trials overall. In addition to those 160 experimental trials, 20 filler items (from the 1- and 10-times tables) were included (Total number of problems = 180). Problems for the filler trials were created following the same procedure as for the experimental trials with one change: b was always 1 or 10.

#### Procedure

In a quiet room, children were assessed on a battery of behavioral assessments in the same fixed order. A “Minion” theme was adopted throughout the testing session, which concluded with the computerized multiplication task during which EEG was recorded (maximum duration: 2.5 h).

##### EEG testing

The same equipment and electrode configuration was used with the children, as described in study 1. However, only the vertical electrooculogram (VEOG) was recorded.

##### Computerized multiplication task

The same equipment and protocol as in Study 1 was used. While the sequence of each trial was the same as in Study 1, to account for developmental differences in arithmetic fluency and prevent the loss of data among slower responders, children were given a longer time to respond to the target solution (maximum: 4,000 ms) (see Fig. 1b). The length of the task was also adapted for the children. They were only assessed on 10 blocks of 18 problems (160 assessed trials, 20 fillers).

### Data analysis

The same analyses were applied on the children’s data as described in Study 1. Due to errors in coding, one unrelated trial was excluded from the children’s EEG data. All remaining experimental trials responded to correctly were segmented (event time window: -200 ms to 4000 ms). After baseline correction (− 200 ms to 0 ms), automatic artifact rejection was performed over a 950 ms interval (− 200 to 750 ms) using the criteria set out by Kappenman et al. (n.d.) [gradient: 75 µV/ms, amplitude: ± 100 µV, minimum–maximum difference: greater than 150 µV/200 ms or less than 0.5 µV/50 ms]. Since residual artifacts were still evident in the VEOG channels of 7 children, the amplitude was altered to ± 75 µV. After excluding trials containing artifacts, an average of 102 trials (74%) remained per child (*SD* = 22; range: 65–143). This consisted of an average of 52 correct trials (*SD* = 12; range: 34–73), 25 related trials (*SD* = 6, range: 14–36), and 25 unrelated trials (*SD* = 6, range 16–36) per child.

**Fig. 4:**
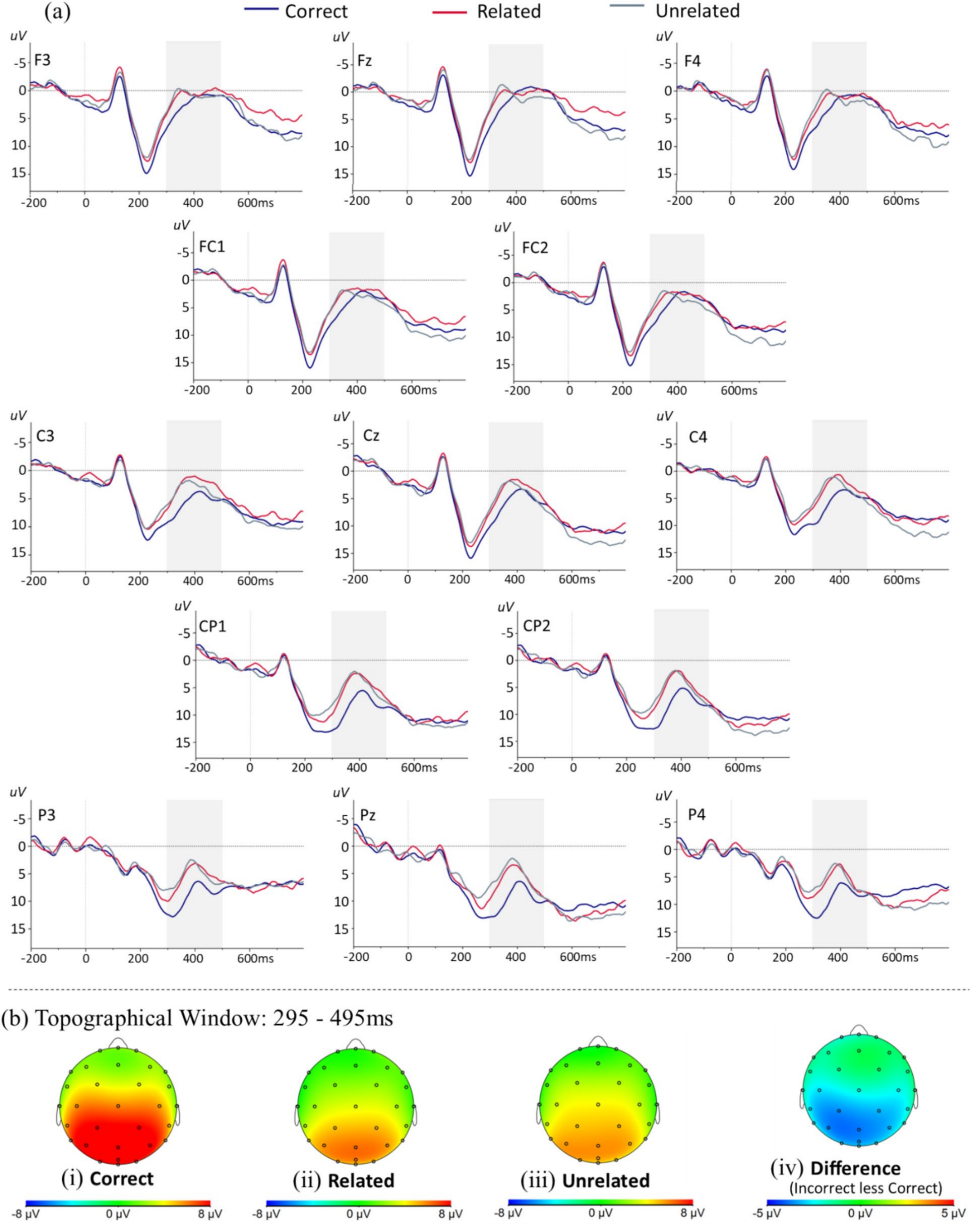
**a** Grand average ERPs on children’s correct (blue), related (red) and unrelated (grey) solutions at electrodes sites across the scalp (negative voltage plotted up). Shaded area highlights the N400 time window (295–495 ms). **b** Topographical plots of children’s grand average for: (i) correct, (ii) related and (iii) unrelated solutions; and (iv) the N400 effect

Visual inspection of the grand-average ERP waveform revealed a prominent negative peak 350 to 450 ms after solution presentation (Fig. 4). This was consistent with an N400 response, rather than the P300 seen in the adult data in study 1. Using a time window of 300–450 ms, the latency of the N400 peak was calculated per participant across all conditions and channels of the grand-average ERP. The mean latency of the N400 peak was identified at 395 ms (*SD* = 18 ms) [correct: 414 ms, *SD* = 19 ms, incorrect: 377 ms, *SD* = 17 ms (related: 383 ms, *SD* = 19 ms, unrelated: 370 ms, *SD* = 15 ms)]. A 200 ms window (295—495 ms) centered around the peak was used to calculate the mean amplitude of the N400 during this time window separately for all children and all conditions.

### Statistical analysis

The same analyses as in Study 1 were repeated on the children’s data. To eliminate extreme scores from RT data, the sequential filtering of the RT data from the computerized multiplication task resulted in a retention of 89% of children’s trials.

A three-way repeated measures ANOVA was run on the mean N400 amplitude, with factors of condition (correct, related, unrelated), hemisphere (left, midline, right) and caudality (anterior, posterior).

## Results

For the descriptive results please see Table 1. A correlation matrix of these outcomes is provided in the Appendix (in Table 4).

### Behavioral data

On the computerized multiplication task, children’s average accuracy was 87.20% (*SD* = 11.51%, range = 58–99%). There was a significant effect of condition on accuracy, *F*(2, 48) = 10.72, *p* < 0.001, *ɳ*^*2*^_*p*_ = 0.31. Accuracy was significantly greater on correct (88.96%, *SD* = 8.36%) than incorrect (83.8%, *SD* = 14.79%) solutions [*F(*1, 24) = 5.99,* p* = 0.022, *ɳ*^*2*^_*p*_ = 0.20], and on the unrelated (88.01%, *SD* = 14.53%) relative to the related (79.86%, *SD* = 16.36%) trials [*F(*1, 24) = 17.86,* p* < 0.001, *ɳ*^*2*^_*p*_ = 0.43].

On average, children took 1103 ms (*SD* = 359 ms, range = 497–1920 ms) to respond to target solutions. A significant effect of condition was evident on RT, *F*(2, 48) = 20.28, *p* < 0.001, *ɳ*^*2*^_*p*_ = 0.46. RTs were significantly faster on the correct (1048 ms, *SD* = 338 ms) than the incorrect (1195 ms, *SD* = 401 ms) solutions [*F(*1, 24) = 25.04,* p* < 0.001, *ɳ*^*2*^_*p*_ = 0.51], and the unrelated (1136 ms, *SD* = 383 ms) than the related (1254 ms, *SD* = 433 ms) solutions [*F(*1, 24) = 14.52,* p* = 0.001, *ɳ*^*2*^_*p*_ = 0.38], see also Appendix, Table .5

Individual differences in children’s accuracy during the arithmetic verification task were significantly correlated with their phonological awareness (*r* = 0.521, *p* = 0.011) and their rate of access^2^ (*r* = -0.566, *p* = 0.005) but not with their verbal working memory (*r* = 0.144, *p* = 0.511). Individual differences in response times did not correlate significantly with any of the phonological measures (see Appendix, Table 6).

### Event related potentials

#### Effect of correctness

A three-way repeated-measures ANOVA on the mean N400 amplitudes revealed a significant main effect of condition *F*(2, 48) = 4.52, *p* = 0.026, *ɳ*^*2*^_*p*_ = 0.16. As expected, the N400 on correct solutions (4.78 µV, *SD* = 3.18 µV) was significantly attenuated relative to incorrect solutions (3.09 µV, *SD* = 3.63 µV). The significant main effect of caudality, *F*(1, 24) = 29.55, *p* < 0.001, *ɳ*^*2*^_*p*_ = 0.55, revealed enhanced N400 amplitudes in anterior (1.97 µV, *SD* = 2.29 µV) compared to posterior (5.34 µV, *SD* = 4.13 µV) ROIs. The difference in amplitude between correct and incorrect solutions was significantly greater in the posterior (difference: 2.85 µV) relative to anterior (difference: 0.52 µV) ROIs, *F*(2, 48) = 11.93, *p* < 0.001, *ɳ*^*2*^_*p*_ = 0.33. The N400 was also significantly modulated by hemisphere, *F*(2, 48) = 4.69, *p* = 0.014, *ɳ*^*2*^_*p*_ = 0.16, with attenuated amplitudes seen at the midline ROIs (4.30 µV, *SD* = 3.28 µV), relative to left hemisphere (3.38 µV, *SD* = 2.96 µV) and right hemisphere (3.29 µV, *SD* = 3.16 µV) ROIs. Finally, the interaction between caudality and hemisphere was significant *F*(2, 48) = 3.25, *p* = 0.047, *ɳ*^*2*^_*p*_ = 0.12. Specifically, the difference in N400 amplitude between anterior and posterior electrode sites was significantly larger (*p* = 0.041) at the midline (difference: 3.84 µV) relative to the right hemisphere (difference: 2.65 µV). No other interactions reached significance.

#### Effect of relatedness

The N400 amplitude did not differ significantly between related (3.15 µV, *SD* = 3.95 µV) and unrelated (3.04 µV, *SD* = 3.32 µV) solutions, neither did the N400 amplitude seen to related and unrelated trials differ by caudality.

The N400 amplitude was neither significantly associated with phonological awareness, nor with rate of access in any of the ROIs (see Appendix, Table 8). However, better performance in verbal WM was positively associated^3^ with the mean amplitude of the related N400 ERP over the left-posterior and right-posterior ROI (Table 3 & Fig. 3). This means that the better a child’s verbal WM, the more attenuated their N400 ERP in response to related solutions in the left- and right-posterior ROIs. None of the correlations for anterior ROIs reached significance.

**Table 3:** Pearson’s correlation coefficients between verbal working memory and the mean amplitude of the N400 ERP to correct, related and unrelated solutions in children across the regions-of-interest after controlling for age and nonverbal reasoning

		Correct			Unrelated			Related		
ROIs		Left	Mid	Right	Left	Mid	Right	Left	Mid	Right
Anterior	*r*	− 0.034	– 0.302	– 0.248	– 0.112	– 0.268	0.130	0.234	0.186	0.246
	*p*	(0.877)	(0.162)	(0.253)	(0.611)	(0.217)	(0.553)	(0.282)	(0.396)	(0.257)
Posterior	*r*	0.332	0.107	0.232	0.230	– 0.090	0.244	0.586*	0.440	0.581*
	*p*	(0.122)	(0.626)	(0.286)	(0.292)	(0.683)	(0.262)	(0.003)	(0.035)	(0.004)

^*^ p < .05 after Benjamini–Hochberg false discovery rate correction

## General discussion

We have presented results from two studies investigating neural indices of arithmetic processing in a sample of adults and a sample of children aged 9–12 years. Participants were required to verify the accuracy of multiplication problems which were either correct or incorrect. Incorrect solutions were either table-related or table-unrelated. Both adults and children were significantly faster when responding to correct than incorrect trials, and an effect of correctness was evident in both groups' EEG data. The ERP wave morphology, however, was characterized by a distinct P300 response in the adult sample, and an N400 response in the child sample. An effect of relatedness was seen in both adults’ and children’s accuracy and reaction time data, but the effect of relatedness was only significant in adults’ ERPs (P300). Finally, individual differences in posterior ERP amplitudes were related to one out of the three measures of phonological processing—verbal WM—in adults and in children, although the nature of this association differed between groups.

### Developmental differences in the neural indices of arithmetic processing

Consistent with recent findings by Grenier et al. (2020), our studies reveal differences between the neural responses of adults versus children during the processing of arithmetic facts. Adult ERPs were characterized by a modulation of the P300, while children’s ERPs demonstrated an N400 modulation. Additionally, the electrode groups at which the ERP modulations were most pronounced differed between age groups, and as a function of condition. Together, these findings contribute to the body of evidence investigating neurocognitive processes supporting multiplication processing as arithmetic expertise develops, although we need to be careful with interpretations due to the limited spatial resolution of EEG.

Beginning with our adult sample, the presentation of the multiplication verification items was followed by a P300 response, with larger amplitudes to correct than incorrect solutions. This result was not in line with our predictions, but similar findings have been reported in recent studies with adult samples (e.g., Dickson & Federmeier, 2017; Grenier et al., 2020; Jasinski & Coch, 2012; Rivera & Soylu, 2021). While the precise neurocognitive processes underlying the P300 response are still debated (e.g., Alday & Kretzschmar, 2019; Polich, 2012), a P300 component is often observed at centro-parietal electrodes following any task requiring stimulus discrimination (Polich, 2007). Within the arithmetic literature, a P300 response has been reported when adults verify visually-presented Arabic digits, with larger amplitudes to correct than incorrect solutions (e.g., Dickson et al., 2018; Jasinkski & Coch, 2012). This has led researchers to conclude that the P300 response may be an index of target detection, when a categorical (yes–no) decision is reached regarding the correctness of a solution (e.g., Dickson et al., 2018; Jasinkski & Coch, 2012).

Our adult results are consistent with this recent literature and add to the emerging literature on the P300 in multiplication verification in adults. In addition to replicating an effect of correctness in adult’s P300 amplitude, our results revealed an interaction between correctness and caudality. Namely, the P300 amplitude to correct solutions was larger at posterior than anterior electrodes, while the P300 amplitude to incorrect solutions was larger at anterior than posterior electrodes. In line with previous research (e.g., Menon et al., 2002; Niedeggen et al., 1999), this suggests that adults rely more heavily on anterior resources when processing incorrect solutions (e.g., Arsalidou & Taylor, 2011; Heidekum et al., 2019; Menon, 2015). Thus, when presented with an incorrect solution, additional top-down cognitive control processes may be necessary to overcome interference, which could explain why a larger P300 amplitude was evident in anterior relative to posterior electrode groups.

In contrast to adults, a modulation of the N400 was evident in children’s ERP responses. In line with our predictions, children showed enhanced N400 amplitudes to incorrect relative to correct solutions. This effect was specific to the posterior electrode groups, and is consistent with previous findings in children (Grenier et al., 2020; Prieto-Corona et al., 2010). Since an N400 response is associated with cognitive processes underlying meaning-making (Kutas & Federmeier, 2011; Kutas & Hillyard, 1980), researchers have proposed that the presence of an N400 might reflect ease of semantic access (Dickson et al., 2022). Given the central role that the parietal cortex plays in the activation and retrieval of multiplication facts, amplitude changes over posterior electrodes would be expected if children were actively retrieving arithmetic facts from semantic memory. Moreover, enhanced amplitudes to incorrect relative to correct solutions might reflect the spreading activation of multiplication solutions in memory networks (Niedeggen & Rösler, 1999; Niedeggen et al., 1999), such that correct solutions are more accessible than incorrect solutions, and consequently result in smaller N400 ERPs over posterior electrodes.

In contrast, the size of the anterior N400 amplitude was comparable across conditions. This is broadly in line with previous studies. For example, Prieto-Corona et al. (2010) reported a significant N400 correctness effect both for anterior and posterior ROIs, but the difference was numerically larger for the posterior region than for the anterior region.

The absence of a P300 in our children’s ERPs could be related to their relative inexperience with multiplication. Indeed, although Dickson et al. (2022) found that children demonstrated a robust N400 response on a multiplication verification task, the beginnings of an adult-like P300 were visible over occipital electrodes, suggestive of a gradual shift to more adult-like ERPs. In adults an N400 modulation (larger for incorrect solutions) instead of a P300 modulation was reported when multiplication operands were presented in an auditory format (Dickson et al., 2018), i.e., in a format that adults were less familiar with. In the same study with adults, when operands were presented visually as Arabic numerals, a P300 was detected (larger for correct targets). Thus, when using a less common auditory format, adults seem to treat stimuli like language and revert to neurocognitive processes associated with meaning-making. Exactly when a P300 response emerges in children’s ERPs requires further examination. Grenier et al. (2020) suggest that this shift has not yet occurred at 11 years-of-age (but see Gómez-Velázquez et al., 2022).

### Relatedness of incorrect solutions does not affect ERPs of novice multipliers

Consistent with previous findings (e.g., Koshmider & Ashcraft, 1991), both adults and children showed an effect of relatedness in their behavioral results. Participants were slower and less accurate at rejecting related than unrelated solutions. This effect was also evident in adults’ but not children’s ERP responses.

When adults responded to incorrect targets, a significant effect of relatedness was found at anterior electrodes, with larger P300 amplitudes for related relative to unrelated solutions. Given that anterior brain regions play an important role in resolving interference, it is perhaps not surprising that related solutions are processed differently from the unrelated solutions at these electrode groups. Yet, if the P300 is an index of decision confidence (Dickson & Wicha, 2019), our behavioral results would predict a smaller P300 for related solutions. Adults, however, showed a larger P300 over anterior electrodes to related than unrelated solutions. The general literature on the P300 (also referred to as the P3, e.g., Polich, 2007, 2012; Snyder & Hillyard, 1976), differentiates between two subcomponents on the basis of topography and function. The fronto-central P3a is believed to reflect early attentional processes in response to novel, unexpected or distracting stimuli; while the parietal P3b is proposed to reflect decision-making, response selection, or contextual updating (Polich, 2007, 2012; Snyder & Hillyard, 1976). From this perspective, our anterior P300 could be categorized as a P3a and a larger P3a would fit with participants needing to recruit additional attentional resources when evaluating related compared to unrelated solutions.^4^

In contrast, we did not find a significant effect of relatedness in children’s ERPs. There are at least two possible explanations. First, given the smaller number of trials available for the related versus unrelated comparison, it is possible that we did not have enough power to detect the presence of an effect of relatedness. Alternatively, there might not be an effect of relatedness on children’s ERP; this absence may be indicative of how children recruit neural networks as relatively novice multipliers. Indeed, similar trends in semantic N400 studies support this latter suggestion. In semantic literature, Benau and colleagues (2011) reported an effect of relatedness in children’s response times, but the electrophysiological results revealed an “all-or-nothing” response to semantically incorrect sentences. That is, children’s N400s were attenuated for congruent compared to incongruent sentences, but the N400 amplitude evoked by moderately and strongly incongruent sentences did not differ. This supports the idea that children might not yet use their top-down control processes differently for related and unrelated solutions.

### Individual differences in verbal working memory are related to posterior ERP amplitudes

Our results show an association between verbal WM and the amplitude of ERPs in posterior ROIs during arithmetic processing. This association was specific to verbal WM and ERPs in posterior ROIs. No correlations between individual differences in the other two phonological subprocesses (rate of access, phonological awareness) and ERP amplitudes in any of the anterior ROIs reached significance. In adults, a stronger verbal WM was associated with a *larger* P300 amplitude on correct solutions across left-posterior and mid-posterior electrodes, as well as with a *larger* P300 amplitude on unrelated solutions in the mid-posterior electrodes. In children, a stronger verbal WM was associated with an *attenuated* N400 amplitude on related solutions in the left-posterior and right-posterior electrodes.

In adult populations, an association between the P300 and WM has previously been reported in studies focusing on non-arithmetic tasks (e.g., Dong et al., 2015; McEvoy et al., 1998; Watter et al., 2001). In the present study, individual differences in verbal WM might also have influenced the allocation of neural resources to process arithmetic problems. Adults with poorer verbal WM capacities may need to recruit additional WM resources to maintain the digits in mind while verifying the accuracy of the proposed solution. In contrast, adults with better verbal WM possibly have a clearer representation of arithmetic facts, or retrieve them more efficiently, especially the easier correct and unrelated solutions.

In our study, children with better verbal WM showed an attenuated N400 response to incorrect related solutions, and is consistent with results from research on the semantic N400. For example, Hampton Wray & Weber-Fox (2013) found that better verbal WM in children was associated with an attenuated N400 response to incorrect items. Our finding of the association of better verbal WM with attenuated N400 responses to incorrect items could thus indicate that children with better verbal WM have better neural access to arithmetic facts, and hence require the activation of fewer neural resources to process the meaning of the problems. Our results suggest that this may especially be the case on the more challenging related solutions. The absence of a significant correlation between behavioral performance measures during multiplication verification (accuracy, response times) and individual difference in verbal WM in children suggests that this neural benefit does not necessarily lead to improved behavioral performance.

Contrary to our findings, Grenier et al. (2020) failed to find a significant relationship between the N400 effect and verbal WM in a large sample of similarly aged children. However, they did not focus on posterior electrodes but analyzed the relationship between verbal WM and the size of the N400 effect (incorrect minus correct) averaged over all electrodes. It is possible that this approach washed out an existing effect specifically found for posterior electrodes.

While neither adults' P300 nor children’s N400 responses were associated with our measures of phonological awareness and rate of access, this does not mean that these phonological processes are not involved in arithmetic processing. Rather, they do not appear to be associated with the electrophysiological responses that we examined in this study. These posterior ERP responses appear to capture neurocognitive processes associated with verbal WM. Interestingly, the significant correlations between behavioral performance measures during multiplication verification (accuracy, response times) and individual difference in phonological awareness and rate of access in children provide further support that multiplication facts are stored as verbal codes in semantic memory.

In summary, our data indicate that individuals with better verbal working memory have a neural advantage during arithmetic fact retrieval. This advantage is evident among primary school-aged children, and is still present once arithmetic fact retrieval has become highly automatized in adulthood. Among children, the advantage is particularly notable when processing incorrect related solutions. However, once arithmetic fact retrieval is highly automatized, the advantage shifts to easier correct and unrelated solutions.

## Conclusion

Our results suggest that when verifying visually-presented arithmetic problems, adults process these problems quickly as targets of categorization, while children process them for meaning. Individual differences in a critical aspect of phonological processing—verbal WM—were related to the P300 amplitude in adults and the N400 amplitude in children at posterior electrodes. Whilst these results highlight that the importance of phonological processing for arithmetic retrieval persists from childhood to adulthood, they also highlight developmental differences in the cognitive and neural processes that underlie multiplication verification.

## Data Availability

No datasets were generated or analysed during the current study.

## Appendix

See Tables 4, 5, 6, 7 and 8.

**Table 4:** Pearson’s correlations of behavioral measures after controlling for age and nonverbal reasoning

	1	2	3	4	5	6
1. Math computation (WRAT-4)	–	0.589**	– 0.286	0.478*	0.182	– 0.347
2. EEG computerized multiplication: accuracy	0.269	–	– 0.162	0.521*	0.144	– 0.566**
3. EEG computerized multiplication: RT	– 0.410	– 0.088	–	– 0.205	0.068	0.363
4. Phonemic awareness	0.087	0.285	0.062	–	0.363	– 0.723**
5. Verbal working memory	– 0.265	– 0.341	– 0.060	0.458*	–	– 0.140
6. Rate of access	– 0.054	0.002	0.216	– 0.499*	– 0.271	–

*Note.* Coefficients below the diagonal relate to adult data, while coefficients above the diagonal relate to child data

^*^ Significant at p < .05

^**^ Significant at p < .01

**Table 5:** Mean (standard deviation) accuracy and RT by trial type on the arithmetic verification task

	Adults (Study 1)		Children (Study 2)	
Trial type	Accuracy (%)	Mean RT (ms)	Accuracy (%)	Mean RT (ms)
Correct	89.58 (5.32)	562 (98)	88.96 (8.36)	1048 (338)
Incorrect	88.71 (5.19)	613 (103)	83.80 (14.73)	1195 (401)
Unrelated	92.10 (4.20)	596 (95)	88.20 (14.51)	1137 (383)
Related	85.67 (6.68)	630 (111)	80.12 (16.38)	1254 (433)

**Table 6:** Pearson’s correlation coefficients between phonological subprocesses and behavioral performance (accuracy & mean reaction time) on the arithmetic verification task (controlling for age and nonverbal reasoning)

	Adults (Study1)		Children (Study 2)	
	Accuracy	Mean RT	Accuracy	Mean RT
Phonological awareness	0.285 (0.211)	0.062 (0.791)	0.521* (0.011)	– 0.205 (0.348)
Rate of access	0.002 (0.992)	0.216 (0.347)	– 0.566** (0.005)	0.363 (0.089)
Verbal working memory	– 0.341 (0.131)	– 0.060 (0.795)	0.144 (0.511)	0.068 (0.757)

^*^*p* < .05, ***p* < .01

**Table 7:** Pearson’s correlations for adults on phonological subprocesses: phonological awareness and rate of access, with the mean P300 ERP amplitude for correct, unrelated and related solutions by regions-of-interest (controlling for age and nonverbal reasoning)

		Anterior ROIs			Posterior ROIs		
Subprocess	Trial type	Left	Mid	Right	Left	Mid	Right
		r (p)	r (p)	r (p)	r (p)	r (p)	r (p)
Phonological awareness ^a^	Correct	– 0.321 (0.145)	– 0.246 (0.269)	– 0.206 (0.358)	– 0.284 (0.200)	– 0.212 (0.344)	– 0.139 (0.538)
	Unrelated	– 0.181 (0.420)	– 0.155 (0.491)	– 0.108 (0.631)	– 0.028 (0.903)	0.096 (0.672)	0.196 (0.383)
	Related	– 0.129 (0.567)	– 0.135 (0.548)	– 0.064 (0.778)	– 0.371 (0.089)	– 0.255 (0.252)	– 0.100 (0.659)
Rate of access ^a^	Correct	0.117 (0.613)	– 0.047 (0.840)	– 0.018 (0.937)	0.033 (0.889)	– 0.053 (0.819)	– 0.089 (0.701)
	Unrelated	0.058 (0.804)	0.006 (0.978)	– 0.060 (0.797)	0.059 (0.799)	– 0.102 (0.661)	– 0.184 (0.424)
	Related	– 0.106 (0.647)	– 0.088 (0.703)	– 0.076 (0.742)	0.058 (0.802)	– 0.017 (0.941)	– 0.082 (0.724)

^a^ Raw Score

**Table 8:** Pearson’s correlations for children on phonological subprocesses, phonological awareness and rate of access, with the mean N400 ERP amplitude for correct, unrelated and related solutions by regions-of-interest (controlling for age and nonverbal reasoning)

		Anterior ROIs			Posterior ROIs		
Subprocess	Trial type	Left	Mid	Right	Left	Mid	Right
		*r (p)*	*r (p)*	*r (p)*	*r (p)*	*r (p)*	*r (p)*
Phonological awareness ^a^	Correct	0.032 (0.886)	– 0.143 (0.516)	– 0.257 (0.237)	0.177 (0.420)	0.003 (0.990)	– 0.024 (0.912)
	Unrelated	– 0.094 (0.669)	– 0.167 (0.447)	– 0.204 (0.350)	0.179 (0.414)	0.053 (0.810)	0.033 (0.882)
	Related	– 0.145 (0.510)	– 0.035 (0.874)	– 0.044 (0.874)	0.113 (0.606)	0.089 (0.685)	0.194 (0.375)
Rate of access ^a^	Correct	– 0.159 (0.468)	– 0.091 (0.680)	– 0.138 (0.529)	– 0.195 (0.372)	– 0.048 (0.828)	– 0.056 (0.800)
	Unrelated	– 0.162 (0.459)	– 0.038 (0.862)	– 0.031 (0.887)	– 0.369 (0.083)	– 0.209 (0.340)	– 0.146 (0.506)
	Related	0.021 (0.923)	– 0.020 (0.927)	– 0.177 (0.419)	– 0.058 (0.798)	0.012 (0.956)	– 0.106 (0.629)

^a^ Raw Score

## Footnotes

1 A reviewer of an earlier version of this manuscript highlighted that the use of the fixation cross might have been problematic. In hindsight, we agree that the choice of a cross which can be confused with a plus sign was not optimal. However, we are confident that this choice did not affect our results for several reasons. First, the fixation cross was present on the screen all the time when there was not a number presented on the screen, i.e., before the problem was presented, after the first operand, after the second operand and after the presentation of the result. Thus, it was clearly not used as a sign indicating an operation. Second, the fixation cross was presented in a much smaller font size than the numerical stimuli. Third, participants had extensive instruction and a large number of practice trials with feedback at the start of the experiment.

2 A larger score on this task is associated with slower rate of access, and therefore, a negative correlation indicates that children who were more accurate on the arithmetic verification task demonstrated better rate of access.

3 Because the N400 ERP is a negative component, a positive correlation indicates that individuals with higher verbal WM show a reduced N400 amplitude.

4 This in turn would suggest that the larger P300 response to correct than incorrect solutions over posterior electrodes, discussed above, might reflect a P3b response.
